# An integrated rehabilitation workforce within secondary healthcare in Pakistan: a qualitative study with physiotherapists

**DOI:** 10.1093/heapol/czaf041

**Published:** 2025-08-13

**Authors:** Kirsty Teague, Shazra Abbas, Aatik Arsh, Dildar Muhammad, Haider Darain, Wesley Pryor, Daniel Llywelyn Strachan

**Affiliations:** Nossal Institute for Global Health, University of Melbourne, Level 2, 32 Lincoln Square North, Carlton 3053, Victoria, Australia; Nossal Institute for Global Health, University of Melbourne, Level 2, 32 Lincoln Square North, Carlton 3053, Victoria, Australia; Institute of Physical Medicine and Rehabilitation, Khyber Medical University, KMU Rd, Phase 5 Hayatabad, Peshawar, Khyber Pakhtunkhwa 25100, Pakistan; Institute of Nursing Sciences, Khyber Medical University, KMU Rd, Phase 5 Hayatabad, Peshawar, Khyber Pakhtunkhwa 25100, Pakistan; Institute of Physical Medicine and Rehabilitation, Khyber Medical University, KMU Rd, Phase 5 Hayatabad, Peshawar, Khyber Pakhtunkhwa 25100, Pakistan; Nossal Institute for Global Health, University of Melbourne, Level 2, 32 Lincoln Square North, Carlton 3053, Victoria, Australia; Nossal Institute for Global Health, University of Melbourne, Level 2, 32 Lincoln Square North, Carlton 3053, Victoria, Australia

**Keywords:** rehabilitation, physiotherapy, workforce, health systems, integration, Pakistan

## Abstract

Understanding how an integrated rehabilitation workforce can be supported and strengthened is crucial to address gaps in access and quality of rehabilitation below tertiary hospitals. We explored how physiotherapists in two provinces in Pakistan perceive enablers and constraints to their rehabilitation performance at individual, workplace, health systems, socio-cultural, and political levels. Using a qualitative approach based on social ecological theories of health-worker performance and semi-structured interviews, 31 in-depth interviews with physiotherapists were conducted at secondary care hospitals in Khyber Pakhtunkhwa and Sindh provinces. Four intersecting themes were generated. (i) The capacity to perform as a rehabilitation professional is mediated by factors operating at different levels of the worker ecology. The experience of these factors has implications for (ii) the livelihoods and wellbeing of rehabilitation workers and (iii) the quality of care these workers perceive is delivered. (iv) Respondents’ insightful and diverse suggestions for positive opportunities for change, towards strengthening and expanding integration of rehabilitation services within the health system, have policy and practice implications. Findings suggest an interdependence between context, rehabilitation workers, and the quality of care they deliver. The perspectives of these workers draw attention, beyond staff numbers and distribution, to the real-world challenges of practicing effectively in the context of local and systemic constraints and facilitators. These insights will be valuable to current efforts to integrate rehabilitation into health care settings beyond tertiary hospitals.

Key messages

- How to improve rehabilitation services integrated into health systems can be better informed by listening to the insights of rehabilitation workers in-post, elucidating context-specific levers for decision-makers to consider.
- Performance of workers, their lived experience of work, and the perceived quality of care provided are affected by factors across the worker ecology, with gendered and provincial differences in the Pakistan context.
- Comparison across two provinces in Pakistan highlighted that integration efforts below tertiary hospitals needs sustained support, particularly governance, management, and cross-sectoral collaboration.

## Introduction

In May 2023, the World Health Assembly (WHA) unanimously adopted a resolution to strengthen rehabilitation services within health systems (World Health Organization 2023). Rehabilitation is ‘a set of interventions designed to optimize functioning and reduce disability in individuals with health conditions in interaction with their environment’ (World Health Organization 2024). Within their resolution, the WHA endorsed rehabilitation as a key component of Universal Health Coverage, while calling for expansion and integration of rehabilitation services at all levels of health systems. To progress this agenda, evidence to inform and drive appropriate decision-making and health system reform is required (Cieza *et al*. 2022, Seijas *et al*. 2023, World Health Organization 2023).

Contextually relevant understanding and solutions to systemic barriers recognised in the literature for integrating rehabilitation in different levels of the health system are currently lacking. These barriers include awareness of rehabilitation disciplines among other health providers, role clarity, resourcing, workloads, unclear referral pathways, poor data, poor availability of relevant rehabilitation expertise, public understanding, and poor governance and political will (Naicker *et al*. 2019, ShahAli *et al*. 2023, Maseko *et al*. 2024).

Many of these barriers point to the performance of rehabilitation workers. Yet, discourse around workforce has primarily focused on labour-market issues (quantity and quality), with minimal health policy and systems research focused on how the rehabilitation workforce functions within these health systems (Naicker *et al*. 2019, Campbell and Mills 2022, Kleinitz *et al*. 2024). Rehabilitation workers offer a first-hand perspective on this critical issue with their insights and commitment likely to be instrumental in any health system integration efforts. This study sought to address these gaps by seeking the perspectives of rehabilitation workers integrated within the specific context of secondary care hospitals in Pakistan. In so doing it aimed to generate important insights regarding rehabilitation workforces integrated beyond tertiary hospitals within the Pakistani health system and guide similar enquiry in other similar settings.

### Setting

Pakistan has a devolved government structure, including health, where each province provides care through a three-tiered system (Muhammad *et al*. 2023). This has enabled different approaches to health workforce employment and deployment in general, including rehabilitation providers in each province (Ur Rehman 2018, Zaidi *et al*. 2019, Muhammad *et al*. 2023). At the time of the study, the provincial systems primarily provided rehabilitation in large provincial hospitals or specialized rehabilitation centres (tertiary care), to a lesser extent through district headquarters (DHQ) hospitals (secondary care), and minimal/no integration at tehsil-level hospitals, rural health centres, and basic health units (primary care). In parallel, there are non-government specialist rehabilitation centres, private clinics, and informal rehabilitation providers. Note that some primary and secondary health services are managed under public–private partnerships (Muhammad et al. 2023).

This study had a specific focus on generating evidence from current rehabilitation workers at secondary care level within two provinces, Sindh and Khyber Pakhtunkhwa (KPK). Physiotherapy is presently the only rehabilitation service provided at the secondary care level of government health systems in both provinces. A wider range of rehabilitation professions (e.g. occupational therapy, prosthetics, and orthotics) are present at tertiary level, non-government organizations, and private practice.

Sindh pioneered training local rehabilitation cadres in Pakistan in 1956 and has a 30-year tradition of physiotherapists operating within secondary healthcare services (Memon *et al*. 2016). By contrast, KPK relatively recently (2014) adopted an initiative to recruit at least one male and one female physiotherapist to each DHQ hospital. They also introduced a governance role for a physiotherapist within the KPK Director General of Health office (Government of Khyber Pakhtunkhwa 2019).

Notably, physiotherapists in both Sindh and KPK have, according to several accounts, campaigned for many years to gain greater professional recognition, autonomy, regulation, and job opportunities (Umar *et al*. 2013, Farhad *et al*. 2014, Memon *et al*. 2016, Ur Rehman 2018, Darain and Arsh 2019). The introduction of a Doctor of Physiotherapy in Pakistan in 2008 was seen as a significant step forward, though a regulatory body remains elusive (Umar *et al*. 2013, Farhad *et al*. 2014, Memon *et al*. 2016, Ur Rehman 2018). Other rehabilitation professions have similarly been advocating for status, with less success, including few reported employment opportunities within government systems (Neelum 2020, Liaqat *et al*. 2021, Mumtaz 2021). This situation has been exacerbated by an increase in private rehabilitation providers over the past decade and a rise in private educational institutions offering unregulated physiotherapy training, leading to greater numbers of physiotherapy graduates whose credentials have been called into question (Memon *et al*. 2016, Gul and Darain 2017, Darain and Arsh 2019).

The perspectives of Pakistani rehabilitation workers regarding health system issues have been documented in four previous research publications (Umar *et al*. 2013, Khan 2019, Liaqat *et al*. 2021, Ahmed *et al*. 2023). However, these studies focused primarily on the tertiary care level, with minimal exploration of rehabilitation workers’ performance, capacity, or integration into the health system. To address these gaps and contribute to emerging global interest in workforce performance among rehabilitation workers, our qualitative enquiry with physiotherapists in KPK and Sindh provinces in Pakistan aimed to understand: (i) what enables and constrains physiotherapists’ work performance, with a focus on the current provision of rehabilitation services at secondary care level; and (ii) what can be done to strengthen rehabilitation within the health systems with a focus on improved integration.

The overarching aim was to generate evidence useful to decision-makers tasked with strengthening current provision and furthering the integration of rehabilitation professions within secondary care level, while expanding rehabilitation services to primary care level.

## Methods

### Design

Qualitative enquiry was used to explore the perspectives of physiotherapists working in DHQ hospitals in KPK and Sindh provinces. Semi-structured in-depth interviews were conducted using a field-tested interview guide (see Interview guide in the online supplementary material). Interviewers’ fieldnotes, daily debriefs during data collection, and weekly team meetings were used to facilitate a reflexive data collection process and as additional sources for data validation through triangulation.

Physiotherapists were the subject of this study because this profession has been the focus of efforts to integrate rehabilitation services below tertiary level within the health system in both KPK and Sindh provinces. A sample of 15–18 participants in each province was targeted. This captured the whole population of eligible physiotherapists in Sindh, and it was considered this would provide reasonable saturation of themes in KPK to allow opportunities for thematic comparison between provinces.

The research team included three team members each from Khyber Medical University, Pakistan (two data collectors and one research lead), and the University of Melbourne, Australia (one project lead, two researchers). All are proficient qualitative researchers, four fluent in Urdu. The two data collectors have advanced experience in qualitative health research (7 and 14 years, respectively). The research team, through a multi-day in-person workshop, codesigned data collection processes, piloted tools and methods, and established collaborative communication used through the research cycle.

Provincial approval to conduct research was granted by the Directors General of Health, KPK and Sindh.

### Theoretical framework

We employed Bronfenbrenner's social ecological theory (Grzywacz and Fuqua 2000, Guy-Evans 2023), a framework originally developed to study the context of child development, but which has since been applied for diverse purposes, including understanding influences on the health workforce, to orient our approach and, in particular, orient our analysis (Ettner and Grzywacz 2001, Baron *et al*. 2014, Lingard and Turner 2015, Kilanowski 2017, Hennein *et al*. 2021).

**Figure 1.**
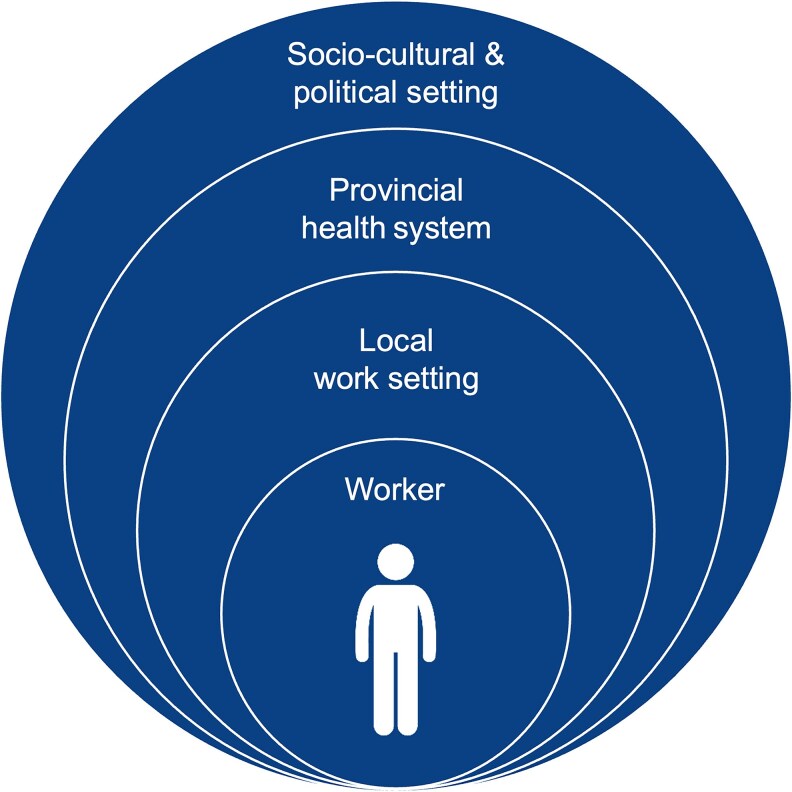
Rehabilitation-worker ecology. Adapted from Bronfenbrenner's social ecological therapy developed for child development (Guy-Evans 2023).

This theoretical framework enabled exploration of the worker ecology that may support or constrain rehabilitation practice at worker, workplace, provincial health system, and socio-cultural and political levels (see Fig. 1).

### Participants

Recruitment was facilitated by the Director General of Health offices in KPK and Sindh, who provided a list of therapists in-post at secondary care level. The invitee list included those who met the inclusion criteria: physiotherapists currently engaged in rehabilitation services in a DHQ hospital in government run KPK or Sindh for at least 1 year. Secondary care facilities under a public–private partnership were not included. In Sindh, where fewer workers are in-post, physiotherapists with long-standing experience at DHQ hospitals, but not currently in-post, were included to enrich data. The list was further refined for maximal sample variation for male and female, rural and urban, length of tenure, and administrative ‘divisions’ within provinces (both provinces have seven divisions containing between 2–9 districts). Due to security concerns, two of the seven divisions within KPK were not covered, nor newly merged districts from the previously Federally Administered Tribal Area. A length of tenure <1 year was considered by in-country researchers to be too short to have the confidence to share reflections on the post, but the variance in insight from those with a fresh perspective to those with longer tenure in-post would be informative. All participants were contacted via WhatsApp and provided with written information prior to interviews by in-country researchers, and oral and written information provided at interview. In Sindh, invitees from three rural districts declined an interview because they were not in their place of duty at that time.

### Data collection and management

Two members of the research team conducted interviews between 16 May and 5 June 2023. All interviewees were invited to choose their preferred venue, with all opting for a private space in their workplace. Both interviewers were males, therefore some female participants chose a colleague to accompany them. The interview guide was used to explore areas of specific interest while remaining sufficiently open to follow lines of enquiry that emerged from respondent testimony (see online supplementary material). Oral consent, including for audio-recording, was sought at the start of the interview and the interviewer signed the consent form to indicate consent received. Interviews were conducted in Urdu for ∼1 h. No material incentives were provided for participation in the research. Interviews were recorded on an audio device.

Following interviews, field notes were written by each interviewer and were uploaded with digital audio recordings to a secure, password-protected, cloud-based storage system, accessible to all researchers. Daily check-ins with data collectors were conducted by a team member in Pakistan and minuted debrief meetings were held with the broader research team. Interviews were transcribed verbatim and translated into English. One data collector and one additional researcher conducted quality checks of both the audio to Urdu transcription and Urdu to English translation.

Data saturation was reached in Sindh, interviewing all available physiotherapists at district level. In KPK the data collector reported approaching data saturation, but that it may not have been met, particularly for sub-themes within the fourth theme ‘opportunities for change’. A pragmatic choice was made to not pursue further interviews in KPK to give parity of numbers for comparison to Sindh and due to security concerns restricting interviews in some areas of the province.

### Data analysis

Verbatim transcriptions of the interviews, field notes, and minutes from debriefs were analysed. Thematic analysis of the interview transcripts was conducted using NVivo^TM^ qualitative analysis software. An initial coding frame was developed based on the key areas of interest set out in the interview topic guide, designed based on the research questions. The coding frame was developed thereafter through an iterative inductive–deductive approach that involved review and amendment of the original frame based on the data generated through the coding process. Key themes were generated using the framework approach to thematic analysis (Pope *et al*. 2000). The data collectors conducted initial coding. One Urdu- and one English-speaking researcher collaboratively reviewed and advanced the coding frame. The broader team provided input at midway and final analysis workshops.

## Results

Interviews were conducted with 16 respondents in Sindh and 15 in KPK, with good coverage across the seven administrative divisions within each province. Rural settings were slightly more represented (58%) and 42% of respondents were female. Average length of tenure was greater in Sindh than in KPK, with recruitment targeting a range of experience levels in each province (Table 1).

**Table 1.** Participant characteristics (*n* = 31).

	Number of interviews	Divisions	Female	Rural	Experience at district level: range (average) in years
Sindh	16	6 of 7	6	8	1–31 (14)
KPK	15	5 of 7	7	10	1–8 (5)

**Figure 2.**
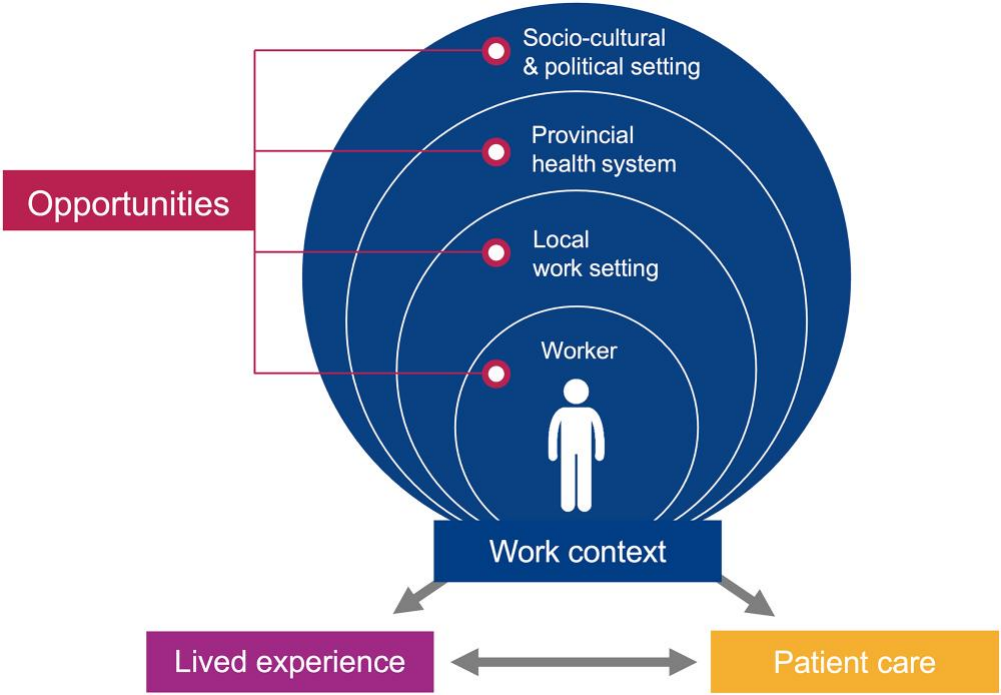
Overarching themes.

Thematic analysis of the 31 interviews explored four overarching themes reflecting how: (i) performance is dependent on elements of ‘work context’ across the worker ecology; (ii) the ‘lived experience’ of work manifests ramifications of the work context for individual workers; (iii) quality of ‘patient care’ is mediated by the work context and lived experiences; and (iv) respondent have insightful suggestions for positive ‘opportunities for change’ towards integration of rehabilitation services within the health system (see Theme map in the online supplementary material). The key findings in and relationship between each overarching theme are summarised in Fig. 2 and detailed below. Each of the four themes cut across all levels of the social-ecological model.

### Work context

This theme captured respondents’ views on the work context and its impact on their role and performance in the context of their immediate work environment, health care system, and socio-cultural and political settings. While some respondents highlighted system-based factors in their work context that enabled role performance, more commonly respondents described local constraints. Some described innovative ways of working that acted as workarounds to constraints, enabling role performance.

Existing governance, management, and conditions of employment were considered to be poorly structured. Respondents considered that support systems and lines of accountability were unclear, and workers lacked a representative voice within governance and management structures.

“In meetings, we are neglected, and our opinions are not sought.” (Female, KPK)

“We can take decisions just to the patient's level instead of other things like funding, etc. … I think the main reason is that nobody supervises us. There must be a leader, higher authority, or someone who can deal with this department, and who can communicate for our field.” (Male, Sindh)

There were exceptions, where others indicated positive structures implemented in their local setting.

“Our hospital has a system where you enter patient data regarding assessment, treatment, and modality. When administrative staff notice the patients’ progress, they recognise our work, and it is the reason they now listen to our problems as well.” (Male, KP)

The respondents experienced inequity in comparison to other forms of health provision and insecurity in conditions of employment.

“When doctors attend a training, the days are not deducted from their casual leave. However, if we [physiotherapists] attend a training, the management deduct the days from our casual leave. This shows a lack of proper support. Why should a therapist be motivated to take part in training or workshops if their leave is negatively affected?” (Male, KP)

Participants explained that due to challenging work conditions in their public sector role, many choose to pursue supplementary private work or consider moving abroad.

“Why [do supplementary private work]? Survival… if the government sector provides a good package, why would we work in the private sector? Look, you work until 2 o'clock here, then you work in the private sector, and after that, you go on private visits. You're human too, but you do it because you have a family to take care of.” (Male, Sindh)

Provision of services were perceived to be impacted by factors related to workload, varied levels of resourcing, and staffing levels. Participants consistently raised the lack of specialist expertise to provide appropriate multidisciplinary rehabilitation care, and insufficient female providers to meet demand from female patients.

“There are no female workers, which is causing problems because when female patients come here for treatment, they request female workers…Yes, it is a major issue. For this, we take help from our female house officers [interns]. This is how work is being done, not properly, but somehow it is continuous” (Male, Sindh)

Additional concerns were raised regarding the provision of rehabilitation in the private sector by physiotherapists and under/unqualified workers. This concern focused on the quality of service provision and the reputational impact for the physiotherapy profession. Prominent contributing factors were perceived to be an oversupply of graduating physiotherapists and the low quality of some institutes.

“… establishing physical therapy institutes in every nook and corner of the country has led to various individuals and households [family businesses] starting their own physical therapy practises. However, there should be some restrictions because, without a proper curriculum, there can be issues related to job competency.” (Female, KPK)

Overall, respondents reported progress with societal understanding of their role. However, they highlighted differences in systems-level progress between the two provinces. Notably, KPK respondents spoke to significant improvements over the past decade, attributed to provincial government investment in male and female posts at DHQ hospitals and a physiotherapy governance role, whereas such progress has not been reported in Sindh.

“If I talk about the district level, I think we are pioneers. I guess 20 PTs [physiotherapists] were at the district level initially. So, the first time when we were appointed as district PT, in the beginning, we faced many problems as we had to set a ground for many things.” (Male, KPK)

“Here, you will find only one to two [physiotherapists] in district hospitals. In most of the districts, there is no physiotherapist. Posts are vacant, and the government is not appointing anyone. No one is looking into this, therefore there is zero improvement.” (Male, Sindh)

### Lived experience of work

This theme captured the effects of the ‘work context’ on individual workers, and particularly how it influenced their self-perception and attitudes towards their work. Respondents shared the varied ways in which they draw value, identity, affirmation, and hope from their profession. They explained situations where these were perceived to be under threat, impacting their motivation in their role, well-being, and mental health.

“Here, you will realise that you are being treated as mere grass. You have no status, no respect” (Male, Sindh)

“You work a lot, but you don't get appreciated for it. You are not called forward. The tasks that were assigned to me as a clinical coordinator were not allowed to be fulfilled, which hurt me a lot. As a result, my energy has naturally decreased. You can understand this.” (Male, Sindh)

However, they also shared instances where progress had been made, especially where the status of their role was perceived to have increased.

“Six years ago, even 18–19 grade officers used to call us malashi [male masseur], and they did not recognise our work. But now, with time and our achievements, they acknowledge us.” (Male, KPK)

There were pronounced differences in the reported experiences of respondents from KPK and Sindh. Relatively recent health system investment in KPK fostered optimistic, hopeful testimony when compared with the relative despondency of respondents from Sindh. Yet increased investment to employ physiotherapists in KPK has introduced nuanced challenges.

“The first reason [behind dissatisfaction with her job] is that physiotherapists should be assigned a job where they belong. For example, I am an Urdu speaker with limited knowledge of Pashto. I don't know about Pashto, so when a patient comes to our department, it's me. I struggle to understand them; I can only grasp their cues, which hinders my ability to assess them accurately or provide proper treatment due to a communication barrier. Secondly, the distance to my workspace is quite far from my home. If I visit my home twice a month, half of my salary is spent on transportation from here to home. Thirdly, it affects my mental health, as living far away from home causes much suffering.” (Female, KP)

There was a prominent gendered difference in the lived experience of work from respondents within KPK, which was absent from the Sindh data. Reports related to a range of concerns from perceived support of colleagues to being heard and valued, being posted far from home, and contributing to patient wellbeing.

“Suppose I share my personal experience as a female physiotherapist. In that case, I know many other female physiotherapists who faced challenges regarding induction in the periphery [outside the capital], environmental factors, and cultural biases. It even goes to the extent of character assassination. Personally, I have suffered a lot due to the above factors. These factors affect not only the professional realm but also impact mental health”. (Female, KPK)

### Patient care

This theme captured respondents’ observations on the influence the ‘work context’ and their ‘lived experience of work’ had on the quality of patient care they perceived their service was able to deliver. Respondents commented on influences at different levels of the worker ecology, including societal norms, systems-level levers, and site-specific factors. Respondents emphasised a perceived lack of recognition from other health workers regarding the role played by physiotherapy and an unhelpful lack of autonomy in patient-level decision-making as particular barriers to the provision of quality patient care.

“When orthopaedic surgeons do the surgery, they do not tell them that exercise is necessary. Then patients come to us to check their range of motion, and by that time, the contracture has occurred already, and no matter how hard we try, our technique does not matter… nothing can fix the range of motion, and young people become disabled, what then does he do?” (Male, KPK)

This lack of professional, collegiate recognition contrasted notably with a perceived increase in recognition among patients of the positive role physiotherapy could play in improving physical health.

“Earlier, there was no awareness of physiotherapy, but now, thank God, people are aware of physiotherapy, that's why I am happy. The patient also started cooperating with us. Earlier, they used to say that physiotherapy is a one-day treatment, but now they have understood that physiotherapy is not a one-day treatment. So, they started cooperating, so it feels good”. (Female, Sindh)

Respondents also identified the availability (or lack thereof) of infrastructure, resources, and staffing as necessary to support and enable the provision of quality care to patients within an appropriate timeframe, with comfort, privacy, and adequate time per patient. Notably, having workspaces where workers could provide appropriate, gender-sensitive care provision was a particular issue.

“This is a district-level setting where accommodating males and females is challenging. People prefer having separate facilities for male and female patients … If we want to provide these services, we cannot do it because males can pass by, and even if we try, female patients may feel uncomfortable, and their families may impose restrictions”. (Female, KPK)

There were several examples where positive change has occurred. The following example demonstrates intersections between our themes, where the respondent perceived increased awareness from the presence of physiotherapy at DHQ level in his setting, and subsequently referrals, with patients reportedly receiving good care. He perceived his role is now valued, with increased agency, and expressed a sense of pride in his department and role.

“But now, at the DHQ level, we properly evaluate and treat the patients through manual therapy, exercises, and modalities. Our hospital physiotherapy department consists of a team of professionals. The hospital staff also attend our department, like doctors and their family members, who witness the positive impact of exercises and therapy physiotherapists provide. This has led to a significant increase in awareness and belief in the effectiveness of physiotherapy as a crucial component of patient treatment. These improvements result in the perception change of others. We are now seen as knowledgeable individuals, and it is no longer the perception that we use machines and provide primary care. The changes in physiotherapy have positively impacted our interactions with patients, and our contribution to their treatment is now better understood and appreciated. This increased level of respect is why patients trust and choose to interact with physiotherapists, recognising the value of their role in the treatment process…with the passage of time, doctors started interacting with us, and they started to understand that prescribing physical therapy is the domain of physiotherapists.” (Male, KP)

### Opportunities for change

This theme captures the range of respondent suggestions for positive systems-level change. Respondents expressed a strong desire for improvement in setting-specific circumstances, an elevation in the status of rehabilitation workers, and bolstered capacity of rehabilitation services in their province toward improved quality of care. Differences between provinces are reflected in the range of suggestions within this theme. Respondents in Sindh advocated for more fundamental changes, such as promotion and recruitment processes, while respondents in KPK called for a more diverse range of reforms including the expansion of services and strengthening of cross-sectoral collaboration.

“To support physiotherapy at the district level, the government should first prioritise the hiring of new physiotherapists based on the advice of senior physiotherapists working in government hospitals in Sindh”. (Male, Sindh)

“The services being provided are good, but if they are expanded to the tehsil level, it would be very beneficial… This would help manage the increasing number of disability cases, such as patients with spinal cord injuries or post-orthopaedic cases with knee contractures, affecting their functionality and daily activities”. (Male, KPK)

Additional suggestions ranged from structural changes through to changes in daily practices, while noting intersections between ways of working and the worker ecology, including patient-centred care and cultural practices and norms.

“It's important to have someone from the local area who understands the cultural norms and can effectively deal with patients according to their cultural background. In physiotherapy, it's not just about performing flexion and extension exercises; the physiotherapy treatment should be tailored according to the cultural context”. (Male, KPK).

Respondents identified a particular need for strengthening governance and management systems across all levels of the health system. They emphasised raising the status of physiotherapists, designating leadership roles for rehabilitation workers, establishing clear lines of accountability, and facilitating parity with other health professions.

“Until there is a regulatory body for the physiotherapy field, people won't be aware, especially in front of the medical community. They still refer to us as technicians. Without a regulatory body, our value is non-existent… Before anything else, our physiotherapists should be valued.” (Female, Sindh)

“The focus should be on taking initial steps to work on areas where there are no facilities provided yet. Secondly, policies should be formulated, followed by the implementation of those policies. After the implementation, there should be proper follow-up to check if the policies are being maintained. The government often opens departments but lacks follow-up and maintenance, leading to a lack of calibre or standard in the long run… we should work together which is the most important thing”. (Male, Sindh)

## Discussion

This study provides insight into what enables and constrains physiotherapists’ ability to deliver rehabilitation services at secondary care level in KPK and Sindh provinces in Pakistan. Importantly, participants in this study explored influences from across their working ecology on their lived experience of work and perceptions of patient care. More specifically, these influences were from their socio-cultural and political environment, their health system, their local workplace, and themselves (i.e. self-perceptions). The intersecting themes generated by the analysis of this study demonstrate that the current working context can have deeply personal impacts on workers and influence patient care. Patient-level interactions were influenced by clinical decision-making autonomy and departmental resourcing, with a gendered difference. Further, participants perceived that demand, access, availability, and utilization of physiotherapy services impacted their ability to provide quality care. Across each of these, there was a substantial influence from social norms, higher education, and the private sector.

The health-system-insider perspectives of physiotherapists captured in this study are timely given the WHA's 2023 Resolution that health systems in low- and middle-income countries expand and strengthen rehabilitation services with an emphasis on integrating rehabilitation as a component of Universal Health Coverage. Despite this, the evidence for operationalizing this integration remains sparce (Campbell and Mills 2022, World Health Organization 2023, Waterworth *et al*. 2024). The following reflections on the findings of this study are presented as a contribution to addressing this gap. Reflections are presented in three key areas: (i) variations in rehabilitation worker testimony across provinces in light of relative health system investment; (ii) the interplay between rehabilitation workers and the regulatory environment, drawing on comparison with regulation of medical professionals in Pakistan; and (iii) rehabilitation worker insights regarding their operational reality in light of the global literature related to worker performance.

### KPK and Sindh: comparative impact of health system investment

Our findings indicate that a decade of investment in rehabilitation at district level has paid dividends in KPK from the perspective of the physiotherapy workforce, while the experience of physiotherapists in Sindh is a reminder that sustained system support is crucial to retain and maintain workforce capacity to sustain decades of investment in rehabilitation services. While acknowledging there has been a shift to public–private partnerships for much secondary level care in Sindh, the role of rehabilitation in these models may warrant further investigation.

Many of the constraints described in the ‘work context’ theme were common to both provincial settings and inclusive of constraints previously published regarding Pakistani rehabilitation workers (Umar *et al*. 2013, Khan *et al*. 2017, Ahmed *et al*. 2023) and broader health professionals (Samad *et al*. 2020, Aman-Ullah *et al*. 2022, Sultan 2022). These primarily relate to poor governance, lack of accountability, conditions of employment, quality control, and resourcing for an overloaded and undervalued workforce. Yet, the differences in how these constraints were experienced by respondents between the two provinces demonstrate the influence of contextual variation in the provincial health system and their impact on integration efforts.

Physiotherapists in KPK recognised the benefits of a recent systematic increase in rehabilitation provision at the secondary care level, with respondents demonstrating a sense of hope, resolve, connection, and empowerment. This was allied to the perceived value afforded them from within and beyond the health system. That there was a governance pathway to influence change in KPK appeared to be a critical aspect in workers’ will and confidence to highlight more nuanced constraints than for respondents from Sindh. In Sindh, while integration of physiotherapy services was ostensibly initiated several decades ago, physiotherapists reported inactivity and neglect from governance structures, and expressed a sense of hopelessness in their role. Some reported that they had fought hard for recognition of their profession over their long tenure, but with minimal perceived success and growing despondency. These findings are consistent with other literature purporting that health worker performance is linked to multiple systems-level levers, but vitally to the ‘humanness’ of this resource, where value is placed on the needs, wellbeing, and insights of workers (Rowe *et al*. 2018, Pangallo *et al*. 2022, Nwankwo *et al*. 2024).

The gendered context of the work environment, as it influences patient care and worker experience, emerged as a salient feature with provincial differentials. Both males and females in KPK and some males in Sindh raised concerns about providing adequate and culturally acceptable care to female patients. Further, all females from KPK raised gender-based issues that negatively impacted their lived experience of work. This was in stark contrast to Sindh, where female respondents did not raise gender-specific issues in the workplace at all. While it may be tempting to equate the prominence of gender issues in KPK to cultural differences between the provinces or to a lack of these issues in Sindh, the findings suggest that raising these issues may instead be a function of the relative investment in targeted employment of female staff at the secondary healthcare level in KPK and increased candour arising from the broader empowerment of the workforce.

These findings suggest that while recruiting and integrating female workers does meet a crucial need, it is not enough. Decision-makers may need to consider gender-specific system-level mechanisms that can facilitate quality care for female patients and promote female worker performance. Previous research within Pakistan has raised a lack of female rehabilitation workers as a barrier to service provision (Naqvi *et al*. 2016, Ahmed *et al*. 2023). However, our findings highlight this as a prominent concern for both male and female physiotherapists. To the best of our knowledge, this is also the first study to report prejudicial experiences faced by female rehabilitation workers in Pakistan, although similar gender-based challenges are documented regarding other female health workers in the country (Abbas *et al*. 2020, Karam *et al*. 2022, Raza *et al*. 2023).

Far from being specific to Pakistan, there is a broad body of global literature that purports that gender bias and disadvantage are systemic issues in rehabilitation, and healthcare more generally, and that this impacts both the female patient and worker (Newman 2014, Stenberg *et al*. 2022, Cullen 2023). However, the hands-on nature of rehabilitation practice, coupled with the social norms of these contexts, that largely disallow one gender from handling another, elevates the importance of addressing this in health system planning in Pakistan. Evidence from this study points to these gender-specific needs of worker and patient, with further enquiry required to provide necessary evidence to inform policy makers.

### Beyond the hospital: key regulatory issues impacting worker performance

The social-ecological framework used in this study revealed a complex interplay between how respondents’ sense of agency, identity, value, role satisfaction, wellbeing, performance, and patient care, met regulatory gaps in both the health and education sectors.

Findings from this study accord with published commentary that an important enabler to professional identity and role satisfaction among physiotherapists in Pakistan has been an increase in status associated with the introduction of a Doctor of Physiotherapy in 2008 (Babur 2015, Memon *et al*. 2016). However, there has subsequently been significant growth in the provision of rehabilitation by un/underqualified practitioners, which goes unchecked without a regulatory body (Ur Rehman 2018). Respondents considered practice by un/underqualified service providers as devaluing their profession, impeding societal understanding of rehabilitation, negatively impacting demand and utilization of their services, and reducing quality outcomes for patients.

Pakistan has a long-term, well documented, tension between qualified and unqualified health professionals, particularly in medical practice (Khan and Mustufa 2023), with accounts emerging within rehabilitation over the last decade (Umar *et al*. 2013, Farhad *et al*. 2014, Shakil-ur-Rehman and Sahibzada 2015). Our findings revealed broad sources of under/unqualified rehabilitation practitioners, ranging from entrepreneurial family members of long-term patients to medical doctors taking on rehabilitation responsibilities, able to practice unhindered. One particularly prominent group of concerns raised by respondents related to a perceived over-supply and unemployment of graduate physiotherapists, and a lack of regulation of the educational institutes producing graduates in Pakistan. This is consistent with the literature reporting an undersupply of rehabilitation professionals in general, but a rapid increase in physiotherapy educational institutions since 2008, with challenges in regulating the quality of these courses and employment opportunities for graduates, with a burgeoning private sector (Gul and Darain 2017, Khan *et al*. 2017, Mumtaz 2021).

The need for a ‘Council’ was a dominant solution raised by respondents for regulation, strongly linked by them to their lived experience, performance, and patient care. Indeed, this solution is a prominent discourse within the physiotherapy community in Pakistan (Farhad *et al*. 2014, Ur Rehman 2018). However, a regulatory body and associated policies in themselves, if not accompanied by implementation of standards and worker supports, may not meet the high expectations of respondents. Medical doctors are regulated in Pakistan, and provinces have specific regulatory policies regarding medical ‘quackery’ (as it is locally coined). Yet, there remain widespread issues with both worker satisfaction amongst doctors and medical ‘quackery’, with a lack of policy enforcement (Khan 2019, Momina and Zakar 2021).

The above serves as a cautionary note regarding the unintended consequences of positive steps forward, such as the introduction of the Doctor of Physiotherapy. It urges those advocating for future gains, such as regulatory bodies, to hold policymakers to account to develop mechanisms for implementing policies and enforcing regulations.

### Improving rehabilitation provision within the health system

This study progresses understanding of some operational elements of integration beyond tertiary hospitals. It highlights the value of the worker as a living component of a health system, with real-time insight and decision-making capacity that has the potential to improve integration efforts. Job creation, distribution, training, and other go-to approaches remain essential, but understanding the operational reality of the worker's experience is a critical component in improving the performance of the integrated workforce (Rowe *et al*. 2018, Nwankwo *et al*. 2024). A recent study from South Africa similarly explored rehabilitation workers’ perspectives on efforts to integrate rehabilitation services below the tertiary setting. Although not focused on worker performance, findings did highlight that workers carry the load of poorly integrated services and have valuable insights into strengthening services (Maseko *et al*. 2024).

Similarly, findings from our research demonstrate the capacity of current rehabilitation workers embedded in the system to generate ideas on positive ways forward for health system reform. Participants ideas were diverse, from facility level (e.g. reallocation of space to enable privacy for female patients), to a broad health systems lens (e.g. the potential for optimizing patient access and outcomes through improved access to rehabilitation at primary care level). Asking, acknowledging, and responding to such insights sends a message that workers are valued, and creates the opportunity to benefit from their willingness to share these insights and participate in positive change (Bandura 2013). The differences in insights and recommendations between respondents from the two provinces seemingly support the notion that engagement of workers, coupled with health system investment, leads to a greater willingness to advocate for positive change. The alternative is seemingly learnt helplessness, where self-efficacy is low and the optimism that any positive outcome will flow from sharing insights is absent (Lennerlöf 2020). Creating opportunities to listen, such as the qualitative methods used in this paper, are important for accessing these valuable insights. The responsibility for responding rests with health sector leadership.

### Study limitations and priorities for future research

This study aimed to capture the experience of rehabilitation workers currently integrated at the lowest level of the health system to-date in KPK and Sindh. In so doing, it limited the study population to physiotherapists or physiotherapy assistants as the only currently integrated rehabilitation workers. In generating important insights this study potentially perpetuates the prevailing norm in Pakistan that sees physiotherapy as synonymous with rehabilitation, when other rehabilitation professionals allegedly struggle to gain traction when advocating for their inclusion—the very first step towards integration—within health systems (Neelum 2020, Liaqat *et al*. 2021). Future research could helpfully capture the perspective of other types of rehabilitation workers more broadly within Pakistan than the two provinces of the current study. Expanding such recruitment should be conducted mindful of sample size for each cadre of workers. In the current study, data saturation was not achieved for all sub-themes, which could potentially be addressed with a larger sample size. Limitations to the number of participants recruited in KPK was largely due to political unrest and security concerns in some areas. Despite this, the insider perspectives provided by physiotherapists operating within the Pakistani health system provide promising evidence and a strong indication of the value of future health policy and systems research regarding rehabilitation workforces in low- and middle-income country settings.

## Conclusion

Pakistan's secondary care rehabilitation workers are committed to patient care but describe complex challenges arising from their working ecology that affect how they provide care and integrate with the broader health system. Comparison of respondent testimony from KPK and Sindh demonstrates both strengths and challenges, while highlighting that integration requires sustained support to retain and maintain staff and services. Revised governance and management structures for services, and regulating practice and education for rehabilitation disciplines, are priorities. Rehabilitation workers provide relevant insights for improvements. Integrating rehabilitation into health systems will benefit from learning and acting on worker experiences and recommendations.

## Supplementary Material

czaf041_Supplementary_Data

## Data Availability

The data underlying this article cannot be shared publicly to preserve participants’ identity, but are available in condensed form on reasonable request to the corresponding author.

## Reflexivity statement

The conceptualization of this study was developed to complement the wider portfolio of work under the United States Agency for International Development ReLAB-HS project by authors from the University of Melbourne, in consultation with ReLAB-HS collaborators in Pakistan. On establishment of the research team, decisions about the design and conduct of this work were shared by all investigators. Two of the authors, including the first, are women. Four of the seven researchers are Pakistani, four have a background as rehabilitation workers, while all have a strong interest and experience in system strengthening. Together the collective expertise and backgrounds of the team informed the interpretation of the data and synthesis of key insights.

## Abbreviations

DHQ: District headquarters
KPK: Khyber Pakhtunkhwa
ReLAB-HS: Rehabilitation Learning Action and Building in Health Systems
WHA: World Health Assembly
