# Supplementary material for: An integrated rehabilitation workforce within secondary healthcare in Pakistan: a qualitative study with physiotherapists
Source: Health Policy Plan. 2025 Aug 13;40(8):920–30. doi: 10.1093/heapol/czaf041 (PMC12448914; doi:10.1093/heapol/czaf041)
Supplement: czaf041_Supplementary_Data [file czaf041_supplementary_data.zip › Teague et al_Supplementary data Interview Guide.docx]

**Supplementary data: Interview Guide**

Implement informed consent procedure and commence recording once respondents have indicated they both understand the purpose of the research and the nature of their involvement and consent to participate.

Relevant demographic information will be collected. Ask and record:

- Place of employment & length of time in the role
- Years of experience
- Tertiary qualification (i.e., Diploma, Masters or Doctor of Physiotherapy. Other qualifications e.g., PhD)
- Specialization and role

Interviewer to discuss the following topics with the interviewee.

- Suggest the interviewer inform the participant the interview asks a series of questions about their work experiences and then later their perspective on workforce in the broader health system.
- Tell me about working as a district level physiotherapist *[Probe: job satisfaction, supports, constraints. Listen and prompt for further detail on elements, as raised, regarding system supports/constraints, the workplace, society and personal]*
- Do you currently work in a different place as well as this role or provide private services at the same facility *[Probe: i.e., private sector. Why? Tell me about that role.]*
- Thank you for telling me about your work. Thinking more broadly, describe to me what is the ideal environment/conditions to optimally perform the role of a rehabilitation worker in [province name]? *[Probe: roles, responsibilities, supports, interactions within and beyond the health system, in the community]*
- What in the [province name] context enables rehabilitation worker effectiveness? *[Probe: relevance at community level, at PHC level, at district level and at tertiary level. Demand side aspects (e.g. community support and supply side aspects (e.g. manageable workload, autonomy, agency of decision making, supervisory support, interactions with other rehab professionals, interactions with community members)]*
- What in the [province name] context constrains rehabilitation worker effectiveness? *[Probe: relevance at community level, at district level and at tertiary level. Demand side issues (e.g. community support and stigma and supply side issues (e.g. workload, autonomy, agency of decision making, supervisory support, interactions with other rehab professionals, interactions with community members)]*
- What are the most urgent and feasible opportunities for positive change to enhance performance in your current role? *[Probe: potential activities, inputs, processes and/or supports to perform their role better]*
- Please explain how the rehabilitation workforce is a part of or linked into the health system? *[Probe: across community, PHC, provincial/district, and national level.]*
- What are the most feasible and urgent opportunities for positive change for the rehabilitation workforce in general in [province name]? *[Probe: barriers and facilitators of change politically and from a governance perspective, financially, culturally, and otherwise specific to the [country name] context]*
- Are there any health workers not currently doing, or informally doing, rehabilitation that you perceive could become/more formally become a part of the workforce providing rehabilitation in [province]? *[Probe: who, what roles, training needs]*
- Are there any other aspects you think it is important to note with regard to what enables and constrains the effectiveness of the rehabilitation workforce in [province name]?

Thank respondent for their time and turn off digital recorder.
