# Supplementary material for: An integrated rehabilitation workforce within secondary healthcare in Pakistan: a qualitative study with physiotherapists
Source: Health Policy Plan. 2025 Aug 13;40(8):920–30. doi: 10.1093/heapol/czaf041 (PMC12448914; doi:10.1093/heapol/czaf041)
Supplement: czaf041_Supplementary_Data [file czaf041_supplementary_data.zip › Teague et al_Supplementary data Theme Map.docx]

**Map to demonstrate relationship between macro and sub codes under the relevant research objectives:**

Research Objective 1:

Understand and document what is enabling and constraining district-level physiotherapists’ work performance with a focus on the current provision of rehabilitation services.

1. WORK CONTEXT
2. Governance and management:
3. Governance & management

- *Local health service*
- *Provincial health system*
- *Physiotherapy profession*

1. Conditions of employment

- *Renumeration*
- *Contracts*
- *Promotions*
- *Posting to outposts*

1. Quality control

1. Rehabilitation workers and workloads

Rehabilitation workforce in their hospital setting

- *Workloads*
- *Coverage of services (inpatient/outpatient)*
- *Physiotherapy Assistants*
- *Use of non-rehab staff*
- *Female therapists*
- *Other rehabilitation professions*

The state of rehabilitation workforce more broadly

- *Supply and quality of graduates*
- *Private service provision*
- *Local healers*
- *Recruitment coverage of the province*
- *Rehabilitation provision below DHQ*

1. Provision of infrastructure and resourcing
2. Physical environment
3. Equipment, machines and maintenance
4. Socio-cultural and political influences:
   1. Change over time
   2. Social and cultural environment
5. LIVED EXPERIENCE
   - 1. Value, identity and hope:

*(exploring how respondents perceive their value and that of their profession to those in their work environment, the health system, and society).*

Support of physiotherapy colleagues

Contributing to patient wellbeing

Heard and valued in the health services

Understood and valued in the community

Professional identity and status

Hope for rehabilitation in the future.

- - 1. Equity and basic needs:

*(describing the personal impact of their conditions of employment and structure of their profession/service in their province).*

Structural inequities

Capacity to participate in change

Meeting basic needs for livelihood

Working in an outpost

- - 1. Experience of the daily work environment:

*(describing the personal impacts of the physical and human work context in their localised workplace).*

1. Workload capacity
2. Being female
3. Physical environment
4. PATIENT CARE
   - 1. Autonomy and patient level decision making:

*(describing how the autonomy the therapist is afforded in the therapeutic space facilitates the capacity to deliver quality care).*

- - 1. Demand and utilisation of the services provided by rehabilitation worker:

*(exploring how respondents perceive people’s understanding, attitudes and interactions with them impacts on demand for services and their capacity to deliver quality care).*

1. Timely care
2. Referral processes
3. Patient expectations and involvement
4. Use of local healers and/or private rehabilitation
   - 1. Physical and human context in delivery of care:

*(exploring how the place and available resources, workforce and infrastructure impacts provision of care).*

1. Time per patient/workloads
2. Availability of other rehabilitation professionals
3. Female staff levels
4. Quality and use of infrastructure
5. Availability and maintenance of equipment and resources

Research Objective 2: Explore and document district-level physiotherapists’ perspectives on strengthening rehabilitation and its integration in the health system and potential activities to improve performance of current and future rehabilitation worker.

1. OPPORTUNITIES FOR CHANGE
   - 1. Improvements in governance of rehabilitation services
        - 1. Local health services
          2. Provincial health system
          3. Physiotherapy profession
     2. Improving capacity for role performance:
        - 1. Changes to broad structure of rehabilitation services

- *Mechanisms for professional regulation and representation*
- *Leadership roles in governance and management structures*
- *Specialisation and role delineation/job descriptions (including assistants and students)*
- *Incorporation of other rehabilitation professionals*
- *Recruitment systems and ratios*
- *Flexibility of processes to locality and culture*
- *Financing*
  - - - 1. Improve condition of employment, particularly where disparities with other health professionals
- *Renumeration*
- *Professional grading/promotions*
- *Job security and contracts*
- *Professional development*
  - - - 1. Improve daily work environment & ways of working
- *Adequate infrastructure, equipment and upkeep*
- *Considerations re female patient and worker*
- *Time per patient*
- *Data collection mechanisms*
- *Communication and decision making on patient care pathway, including referral mechanisms within and between institutions*
  - 1. Expansion and cross sectoral strengthening:

1. Expansion of rehabilitation services to lower levels of the health system
2. Address the rehabilitation education sector (quality and quantity of institutions and graduates)
3. Address the awareness of rehabilitation within and beyond the health system
4. Roles for non-rehabilitation workers in rehabilitation pathways
